# Health Benefits from Large-Scale Ozone Reduction in the United States

**DOI:** 10.1289/ehp.1104851

**Published:** 2012-07-18

**Authors:** Jesse D. Berman, Neal Fann, John W. Hollingsworth, Kent E. Pinkerton, William N. Rom, Anthony M. Szema, Patrick N. Breysse, Ronald H. White, Frank C. Curriero

**Affiliations:** 1 Department of Environmental Health Sciences, Johns Hopkins Bloomberg School of Public Health, Baltimore, Maryland, USA; 2 Office of Air Quality Planning and Standards, U.S. Environmental Protection Agency, Research Triangle Park, North Carolina, USA; 3 Environmental Health Policy Committee of the American Thoracic Society, Washington, DC, USA; 4 RH White Consultants LLC, Silver Spring, Maryland, USA; 5 Department of Biostatistics, Johns Hopkins Bloomberg School of Public Health, Baltimore, Maryland, USA

**Keywords:** health benefits, health impact assessment, ozone, standards

## Abstract

Background: Exposure to ozone has been associated with adverse health effects, including premature mortality and cardiopulmonary and respiratory morbidity. In 2008, the U.S. Environmental Protection Agency (EPA) lowered the primary (health-based) National Ambient Air Quality Standard (NAAQS) for ozone to 75 ppb, expressed as the fourth-highest daily maximum 8-hr average over a 24-hr period. Based on recent monitoring data, U.S. ozone levels still exceed this standard in numerous locations, resulting in avoidable adverse health consequences.

Objectives: We sought to quantify the potential human health benefits from achieving the current primary NAAQS standard of 75 ppb and two alternative standard levels, 70 and 60 ppb, which represent the range recommended by the U.S. EPA Clean Air Scientific Advisory Committee (CASAC).

Methods: We applied health impact assessment methodology to estimate numbers of deaths and other adverse health outcomes that would have been avoided during 2005, 2006, and 2007 if the current (or lower) NAAQS ozone standards had been met. Estimated reductions in ozone concentrations were interpolated according to geographic area and year, and concentration–response functions were obtained or derived from the epidemiological literature.

Results: We estimated that annual numbers of avoided ozone-related premature deaths would have ranged from 1,410 to 2,480 at 75 ppb to 2,450 to 4,130 at 70 ppb, and 5,210 to 7,990 at 60 ppb. Acute respiratory symptoms would have been reduced by 3 million cases and school-loss days by 1 million cases annually if the current 75-ppb standard had been attained. Substantially greater health benefits would have resulted if the CASAC-recommended range of standards (70–60 ppb) had been met.

Conclusions: Attaining a more stringent primary ozone standard would significantly reduce ozone-related premature mortality and morbidity.

Tropospheric ozone is a secondary air pollutant that has been recognized as a serious public health risk [U.S. Environmental Protection Agency (EPA) 2008c]. Tropospheric ozone has been associated with adverse health effects, including decreased pulmonary function, asthma exacerbations, increased hospital and emergency department (ED) visits, and increased mortality (Choi et al. 2011; Dockery and Pope 1994; Levy et al. 2001; Mudway and Kelly 2000). Evidence of negative health effects has been demonstrated by toxicological studies (Larsen et al. 2010), clinical trials (Gong et al. 1986), longitudinal epidemiological studies (Bell et al. 2004; Gryparis et al. 2004; Huang et al. 2005; Ito et al. 2005; Levy et al. 2005; Schwartz 2005), and cohort epidemiological studies (Jerrett et al. 2009). Adolescents and individuals with existing chronic lung and cardiovascular disease have increased susceptibility to the adverse health effects of ozone (Burnett et al. 2001).

The Clean Air Act (CAA 1970) mandated health-based national ambient air quality standards (NAAQS) set at a level “requisite to protect public health with an adequate margin of safety.” Health impact assessments (HIAs) are used to make informed and systematic decisions about regulatory policy based on estimates of positive and negative health impacts from proposed air pollution standards (Briggs et al. 2009). To systemize health impact estimations associated with air pollution, the U.S. EPA Office of Air Quality Planning and Standards uses the software tool Environmental Benefits Mapping and Analysis Program (BenMAP; Abt Associates Inc., Bethesda, MD) (U.S. EPA 2010a). The BenMAP interface can be used to predict changes in air pollution, quantify exposed populations, and estimate changes in health outcomes according to geographic location within the United States. BenMAP (U.S. EPA 2010a) has been applied to inform local and federal regulatory policies concerning exposure to ozone (Hubbell et al. 2005; U.S. EPA 2008b), PM_2.5_ (Davidson et al. 2007; Fann et al. 2011), and diesel exhaust (Connecticut Department of Environmental Protection 2009), to simulate climate change scenarios (Tagaris et al. 2010; Voorhees et al. 2011), and to conduct international assessments (Boldo et al. 2011).

As required under the CAA (1970), the U.S. EPA Clean Air Scientific Advisory Committee (CASAC) performs a formal review of scientific literature and provides advice to the U.S. EPA Administrator on the adequacy of the NAAQS. Based on its 2008 evidence review, the CASAC recommended adoption of a primary 8-hr average ozone standard in the 60- to 70-ppb range. A 2009 U.S. EPA research assessment reviewed ozone exposure and the respiratory-related mortality evidence, including a pertinent long-term cohort study of 96 metropolitan statistical areas (MSAs) that estimated a 1.04 [95% confidence interval (CI): 1.01, 1.07] relative risk in cardiovascular mortality for each 10-ppb increase in ozone (Jerrett et al. 2009). In 2009, the U.S. EPA initiated a reconsideration of the 2008 ozone NAAQS and in 2010 proposed a revised standard in the range from 60 to 70 ppb for an 8-hr daily maximum (U.S. EPA 2010b). However, on 2 September 2011 the President of the United States requested the U.S. EPA Administrator defer review of the ozone NAAQS to 2013, citing concerns about regulatory uncertainty (Office of the Press Secretary 2011).

Recent HIAs have estimated the expected human health benefits of attaining a more stringent ozone NAAQS in the future (Fann et al. 2011; U.S. EPA 2008b). The goal of the present analysis was to evaluate immediate health benefits that would result at nationwide and regional levels from meeting the current NAAQS ozone standard of 75 ppb, and two more stringent standards of 70 and 60 ppb. We used recent ozone monitor data and current concentration–response (C-R) function estimates to estimate changes in ozone-related premature mortality and morbidity that would have been observed during 2005–2007 instead of projecting data into the future. We hypothesized that ozone-related premature mortality and morbidity would have decreased across the United States, with regional variability across the nation.

## Methods

*Data sources: air pollution and risk estimates.* We acquired ozone monitoring data for 2005–2007, the most recent data years available in BenMAP, from the U.S. EPA’s Air Quality System (AQS) regulatory air pollution monitor network (U.S. EPA 2012a). Monitoring data were supplied by federal, state, local, and tribal air pollution control agencies and audited by the U.S. EPA for quality assurance, including sampler performance, precision, bias, and accuracy (U.S. EPA 2008a).

We examined epidemiological literature for ozone-attributed C-R functions for mortality- and respiratory-related morbidity to summarize the association between ozone concentration and health (Table 1). Because our study focuses on regulatory policy with ozone, we used guidance from a recent U.S. EPA ozone regulatory impact assessment to select studies that maximize national applicability (U.S. EPA 2008b). The selection criteria included an array of statistical designs and broad geographic coverage. We chose time-series studies (Bell et al. 2004; Huang et al. 2005), meta-analyses (Bell et al. 2005; Ito et al. 2005; Levy et al. 2005), a case-crossover study (Schwartz 2005), and a cohort study of long-term effects (Jerrett et al. 2009). Morbidity studies were primarily time-series analyses although they included fewer cities and years than mortality studies. Except for ED visits, the morbidity estimates were limited to age-specific populations. We felt that of the substantial ozone literature available, these selected C-R functions represent a small but well-cited and diverse subsection of critical ozone health effects estimates.

**Table 1:** Epidemiological studies used as a source of C-R function data. Morbidities were pooled to estimate health effects.

Health end point	Reference	Study design
Nonaccidental mortality	Bell et al. 2004	Time-series of short-term ozone over 14 years; 95 U.S. cities (NMMAPS); all ages. Distributed lag model (community rates); hierarchical model (national rate).
Ito et al. 2005	Meta-analysis (43 studies) of short-term ozone (global); additional U.S. city time-series (n = 7); all ages.
Schwartz 2005	Case-crossover of short-term ozone over 8 years; 14 U.S. cities; all ages. City-specific regression (community rates); iterative MLE (national rate).
All-cause mortality	Bell et al. 2005	Meta-analyses (39 studies) of short-term ozone; NMMAPS time-series; all ages. Two-stage Bayesian hierarchical model.
Levy et al. 2005	Meta-analyses (71 time-series reviewed; 28 selected) of short-term ozone; all ages. Hierarchical linear model.
Cardiopulmonary mortality	Huang et al. 2005	Time-series of short-term ozone; 19 U.S. cities (NNMAPS); all ages. Hierarchical distributed lag model.
Respiratory mortality	Jerrett et al. 2009	Cohort study of long-term ozone effects for 96 MSAs over 18 years; > 30 years of age. Multilevel Cox regression models.
Hospital admissions (respiratory disease)	Schwartz 1995	Time-series of short-term ozone over 3 years. 2 U.S. cities; elderly (> 65 years of age). Poisson regression model.
Burnett et al. 2001	Time-series of short-term ozone exposure over 3 years; Toronto, Canada; children < 2 years of age. Log relative risks estimated with an exponential function.
Hospital admission [chronic lung disease, pneumonia, lung disease (minus asthma)]	Schwartz 1994	Time-series short-term ozone exposure over 3 years; Detroit; elderly (> 65 years of age). Poisson regression model.
School-loss days	Chen et al. 2000	Time-series of short-term ozone over 3 years; children (5–17 years of age).
Gilliland et al. 2001
Acute respiratory symptoms (restricted activity)	Ostro and Rothschild 1989	Time-series of short-term ozone over 5 years; Urban United States; adults (18–65 years of age). Poisson regression model.
Asthma-related ED visits	Jaffe et al. 2003	Time-series of short-term ozone over 5 years; 3 Ohio cities; young people (5–34 years of age). Poisson regression model.
Peel et al. 2005	Time-series of short-term ozone over 7 years; Atlanta; all ages. Poisson generalized estimating equation model.
Wilson et al. 2005	Time-series of short-term ozone over 3 years;2 U.S. cities; all ages. Generalized additive model.
Abbreviations: MLE, maximum likelihood estimation; NMMAPS, National Morbidity, Mortality, and Air Pollution Study.				

*Air pollution monitoring, rollback, and interpolation.* We selected air monitors according to the NAAQS criteria (U.S. EPA 2008c). Data were restricted to the 153-day ozone season (1 May–30 September) and monitors with at least nine hourly observations for > 50% data days. Because monitors are sometimes collocated and measure multiple pollutants, the U.S. EPA uses parameter occurrence codes (POCs) to distinguish primary devices, so we chose only monitors with a POC ≤ 4 for inclusion (U.S. EPA 2008a).

We assumed a policy-relevant background level for non-anthropogenic ozone of 40 ppb, which is the upper limit of average background ozone during the warm season based on a GEOS-Chem model of the United States (Wang et al. 2009). To assess the impact of regulatory changes, we “rolled back” (i.e., reduced) air monitor concentrations to achieve the hypothetical ozone standards. Specifically, we used a quadratic rollback approach that proportionally reduced ozone levels such that simulated concentrations at any one monitor were reduced more during high ozone days than lower ozone days. This approach affects concentrations substantially above the regulatory limit more than those near compliance (Rizzo 2005). Rounding to two significant digits, we added 0.9 ppb to each standard while initiating compliance at the fourth highest daily ozone value to mitigate the influence of anomalously high measurements. We interpolated ozone to a 36 × 36-km grid using the deterministic Voronoi Neighbor Averaging (VNA) technique, a default BenMAP option (Chen 2004; Hubbell et al. 2005: U.S. EPA 2010a).

In addition to quadratic ozone rollbacks, we performed sensitivity analyses assuming a “peak shaving rollback” in which ozone concentrations above the systemic compliance cap were truncated, but ozone levels below the cap were left at measured levels. We also performed sensitivity analyses assuming alternative background concentrations (e.g., 20 and 30 ppb) and evaluated the impact of altered ozone levels when applied across a full year, instead of limiting the analysis to a warm season.

*Estimation of health impacts.* Health impact functions enable the quantification of health outcomes (e.g., ozone-related mortality and morbidity) from changes in population exposure to air pollution. A log-linear function contains four components and has the basic form of

Δ*y* = *y*_0_(e^βΔ^*^x^* – 1)*Z*,

where *y*_0_ is the baseline incidence rate of the health outcome, β is the coefficient of association between ozone concentration and health outcome (i.e., the C-R function based on epidemiological research), Δ*x* is the estimated air pollution change (i.e., the difference between the current interpolated ozone level and the interpolated ozone level assuming attainment of the NAAQS), *Z* is the size of the exposed population, and Δ*y* is the estimated change in the health outcomes due to the change in ozone exposure. This approach assumes a log-linear C-R function across the entire range of possible ozone concentrations, such that effects may be observed below NAAQS levels (Bell et al. 2006).

We used morbidity and mortality C-R functions reported for daily 8-hr maximum ozone exposures, or adjusted the reported C-R functions to be consistent with daily 8-hr maximum exposures using national conversion factors (e.g., if based on 24-hr mean or 1-hr maximum ozone concentrations) (Thurston and Ito 2001). In addition, we used C-R functions derived using warm-season data (May–September). Using multicity mortality studies provided better control for the confounding effects of particulate matter and gaseous pollutants, including carbon monoxide (CO), nitrogen dioxide (NO_2_), and sulfur dioxide (SO_2_) (Bell et al. 2004; Thurston and Ito 2001).

U.S. Census data for 2000 were aggregated from census blocks to a 12 × 12-km grid and extrapolated to estimate population sizes in 2005, 2006, and 2007 using growth factors. [For estimated population sizes used for each outcome and year, see Supplemental Material Table S1 (national estimates) and Table S2 (MSAs used to derive regional estimates) (http://dx.doi.org/10.1289/ehp.1104851).] Morbidity incidence rates are U.S. EPA estimates from national and local data sources, including the Centers for Disease Control and Prevention (Atlanta, GA), National Center for Health Statistics (Hyattsville, MD), Health Care Cost and Utilization Project (Rockville, MD), National Center for Education Statistics (Washington, DC), and individual studies (U.S. EPA 2010a). We estimated exposures at the grid level and assumed that all individuals within a grid cell experienced the same changes in exposure levels. We estimated changes in health outcomes at the county level according to the fraction of population-level grids that fell within county boundaries.

Because C-R functions for morbidities were derived from fewer cities and smaller data-year sample sizes, we pooled risk estimates for the same outcome across studies using a random-effects weighting procedure to derive a single C-R function. Specifically, we used a two-stage inverse variance weighting approach to account for both between-study variability and within-study individual effect estimates that minimize uncertainties based on spatial and temporal data availability (Hubbell et al. 2005). For respiratory-related ED visits, pooling increases the data by combining studies limited to younger individuals (Jaffe et al. 2003) with studies that cover all ages (Peel et al. 2005; Wilson et al. 2005). We used Monte Carlo simulation from the pooled risk estimates to generate 90% confidence intervals based on the C-R function variance.

*Data reporting and software.* Expected changes in ozone concentrations from meeting the regulatory standards were mapped and used to estimate avoided morbidities and mortalities for the United States (excluding Alaska and Hawaii). In addition, we estimated reductions in nonaccidental mortality for the 15 most populous MSAs using the C-R function derived by Bell at al. (2004).

The HIA was performed using the U.S. EPA’s publicly available BenMAP software (version 4.0.43; U.S. EPA 2012b). We created maps using ESRI® ArcMAP^™^ software (version 10.0; ESRI, Redlands, CA) and summarized results with the R Statistical Computing Environment (version 2.11; R Project for Statistical Computing, Vienna, Austria).

## Results

*Air monitor data.* A total of 1,170, 1,168, and 1,183 AQS ozone air monitors fit the data completeness criteria for 2005, 2006, and 2007, respectively. Mean values for daily 8-hr maximum ozone warm-season measurements ranged from 44.9 to 45.5 ppb (Table 2). From visual observation, the highest regional concentrations appeared to be in noncoastal southern (53.6 ppb) and central (52.8 ppb) portions of California, near Salt Lake City (55.6 ppb), southern Arizona (51.7 ppb), and portions of western North Carolina (55.1 ppb). Moderately elevated concentrations were observed throughout the Midwest, South, the Northeast corridor, and Texas.

**Table 2:** Summary of 8-hr maximum ozone measurements (ppb) averaged across eligible AQS monitors.

Summary value	2005	2006	2007
Mean	45.13	44.90	45.51
Minimum	20.31 (Alameda, CA)	26.23 (Sonoma, CA)	24.70 (King, WA)
Maximum	62.11 (Maricopa, AZ)	65.57 (El Dorado, CA)	68.94 (San Bernardino, CA)
SD	6.35	6.07	6.93
Count	1,170	1,168	1,183
Counties indicate locations of minimum and maximum observed values.						

**Figure 1:**
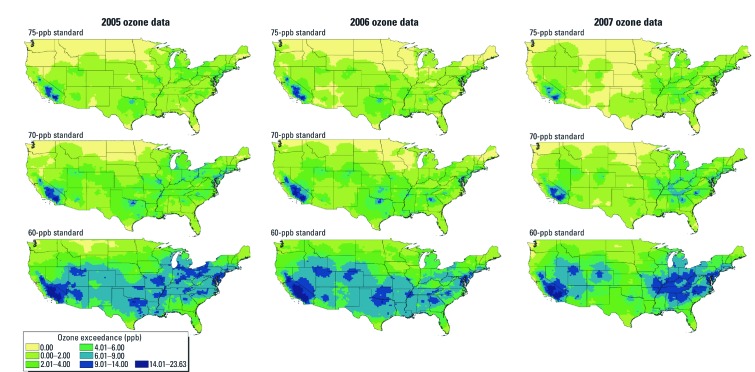
Estimated reductions in annual ozone (8-hr maximum) if regulatory attainments of 75, 70, and 60 ppb had been achieved (2005–2007).

*Interpolated ozone rollbacks.* Estimates of ozone reduction following the proposed regulatory standards are presented for the United States (Figure 1). Considerable temporal variation within locations could be observed by year. For 2006, the estimated mean ozone reduction is 0.97 ppb at the 75-ppb standard, 2.06 ppb at the 70-ppb standard, and 5.73 ppb at the 60-ppb standard. The maximum estimated ozone reduction for 2006 is 23.63 ppb under a 60-ppb standard in southern California. Values estimated using 2005 and 2007 data are slightly different, but spatial patterns were generally consistent across years. There is substantial geographic variation, with the highest estimated reductions occurring in southern California, the Midwest, and portions of the Northeast corridor.

*Nationwide mortality and morbidity health impacts.* Annual avoided premature mortalities were estimated for the United States (Table 3). Under the current 75-ppb standard, the greatest reductions in all-cause mortalities were estimated for 2005, ranging from 2,480 (90% CI: 1,830, 3,130) using the C-R function derived by Levy et al. (2005) to 1,760 (90% CI: 990, 2,530) using the Bell et al. (2005) estimate. We estimated fewer avoided deaths in 2006 (640 cardiopulmonary mortalities at the current standard), the year with the smallest mean ozone concentration, compared with 2005 and 2007 (800 and 700 avoided cardiopulmonary-related mortalities at the current standard). Estimates of reductions in nonaccidental deaths were smallest when based on the C-R function derived from Bell et al. (2004), and largest based on the C-R function from Ito et al. (2005) (e.g., 900 fewer deaths (90% CI: 400, 1,400) compared with 2,780 (90% CI: 1,420, 5,000) in 2005 under the 70-ppb standard, respectively). We estimated that there would be almost three times as many respiratory mortalities (1,970) avoided than cardiopulmonary-related deaths (700) at the 75-ppb standard for 2007 with similar trends for other years. As expected, estimated numbers of avoided health outcomes increased as proposed regulatory levels became more stringent. For example, using the C-R function from Levy et al (2005) and data for 2005, we estimated 2,480 avoided deaths if the current 75-ppb standard had been met, compared with 4,130 avoided deaths under a 70-ppb standard (a 67% increase in avoided deaths) and 7,990 avoided deaths (a 222% increase) under a 60-ppb standard. This general trend holds true across data years and alternative risk estimates.

**Table 3:** Estimates of nationwide prevented mortalities (mean and 90% CI) after meeting the current and proposed ozone regulatory standards (2005–2007).^a,b^

75-ppb standard			70-ppb standard			60-ppb standard		
Health end point	2005	2006	2007	2005	2006	2007	2005	2006	2007
Nonaccidental mortality
Bell et al. 2004	540 (240, 840)	430 (190, 670)	470 (207, 730)	900 (400, 1,400)	750 (330, 1,160)	822 (360, 1,280)	1,740 (770, 2,710)	1,590 (700, 2,480)	1,690 (750, 2,640)
Ito et al. 2005	1,670 (850, 3,010)	1,340 (680, 2,400)	1,460 (740, 2,630)	2,780 (1,420, 5,000)	2,320 (1,180, 4,170)	2,550 (1,300, 4,590)	5,380 (2,750, 9,670)	4,930 (2,520, 8,860)	5,250 (2,680, 9,430)
Schwartz 2005	820 (350, 1,300)	660 (280, 1,040)	720 (300, 1,130)	1,370 (580, 2,150)	1,140 (480, 1,800)	1,250 (530, 1,970)	2,640 (1,120, 4,170)	2,420 (1,020, 3,820)	2,580 (1,090, 4,070)
All-cause mortality
Bell et al. 2005	1,760 (990, 2,530)	1,410 (790, 2,030)	1,540 (860, 2,210)	2,930 (1,650, 4,210)	2,450 (1,380, 3,520)	2,690 (1,510, 3,860)	5,680 (3,190, 8,160)	5,210 (2,930, 7,490)	5,540 (3,110, 7,950)
Levy et al. 2005	2,480 (1,830, 3,130)	1,990 (1,470, 2,510)	2,160 (1,600, 2,730)	4,130 (3,040, 5,210)	3,450 (2,550, 4,360)	3,780 (2,790, 4,780)	7,990 (5,900, 10,100)	7,330 (5,410, 9,250)	7,790 (5,750, 9,830)
Cardiopulmonary mortality	800 (380, 1,210)	640 (310, 980)	700 (330, 1,060)	1,320 (630, 2,010)	1,110 (530, 1,680)	1,210 (580, 1,850)	2,550 (1,220, 3,880)	2,340 (1,120, 3,560)	2,490 (1,190, 3,790)
Respiratory mortality	2,230 (1,000, 3,450)	1,790 (800, 2,770)	1,970 (880, 3,050)	3,730 (1,670, 5,770)	3,110 (1,390, 4,810)	3,440 (1,540, 5,320)	7,210 (3,250, 11,100)	6,620 (2,980, 10,200)	7,060 (3,180, 10,900)
aReferences for morbidity C-R estimates are provided in Table 1. bPopulation sizes used to derive these estimates are shown in Supplemental Material, Table S1 (http://dx.doi.org/10.1289/ehp.1104851).												

Avoided morbidities were variable with large numbers of school-loss days and acute respiratory symptoms (Table 4). Across the data years, acute respiratory symptoms would have been reduced by 3–3.5 million cases/year if the current 75-ppb standard had been achieved, and 10.3–11 million acute respiratory symptom events prevented under a 60-ppb standard, representing a 300% increase in avoided outcomes. In addition, we estimate that > 1 million school-loss days would have been avoided if the current standard had been met, which highlights the impact of ozone on younger individuals. The large confidence intervals for estimated morbidity counts are partially driven by the pooling of multiple C-R functions.

**Table 4:** Estimates of nationwide prevented morbidities (mean and 90% CI) after meeting the current and proposed ozone regulatory standards (2005–2007).^a,b^

75-ppb standard			70-ppb standard			60-ppb standard		
Health end point	2005	2006	2007	2005	2006	2007	2005	2006	2007
Acute respiratory symptoms	3,567,000 (1,821,000, 5,304,000)	3,016,000 (1,541,000, 4,482,000)	3,070,000 (1,567,000, 4,566,000)	5,834,000 (2,983,000, 8,666,000)	5,034,000 (2,574,000, 7,477,000)	5,273,000 (2,695,000, 7,835,000)	11,086,000 (5,688,000, 16,426,000)	10,305,000 (5,285,000, 15,274,000)	10,655,000 (546,400, 15,795,000)
ED visits (respiratory)	1,800 (0, 4,030)	1,460 (0, 3,250)	1,500 (0, 3,350)	2,920 (1, 6,520)	2,450 (1, 5,450)	2,590 (1, 5,740)	5,570 (1, 12,600)	5,070 (1, 11,400)	5,260 (1, 1,1800)
Hospital admissions (respiratory)	1,310 (330, 2,510)	1,000 (250, 2,000)	1,150 (300, 2,200)	2,220 (570, 4,260)	1,790 (440, 3,500)	2,040 (530, 3,900)	4,280 (1,110, 8,230)	3,810 (960, 7,460)	4,150 (1,080, 7,960)
School-loss days	1,241,000 (553,000, 1,999,000)	1,051,000 (468,000, 1,693,000)	1,036,000 (461,000, 1,667,000)	2,022,000 (901,000, 3,259,000)	1,740,000 (775,000, 2,805,000)	1,775,000 (791,000, 28,610,000)	3,841,000 (1,716,000, 6,209,000)	3,540,500 (1,581,600, 5,721,200)	3,591,000 (1,604,000, 5,802,000)
aReferences for morbidity C-R estimates are provided in Table 1. bPopulation sizes used to derive these estimates are provided in Supplemental Material, Table S1 (http://dx.doi.org/10.1289/ehp.1104851).												

Results from sensitivity analyses demonstrated that reducing the assumed non-anthropogenic background ozone level (i.e., the policy relevant background level) from 40 to 20 ppb would increase estimated numbers of avoided nonaccidental deaths by about 10% nationally (data not shown). Estimated numbers of avoided deaths were approximately 66% lower when we used a peak shaving approach to estimate reductions in ozone levels compared to the default quadratic rollback (data not shown). Because peak shaving assumes that ozone levels are only reduced at times and locations when air monitors indicate levels above the standard, it does not provide a realistic representation of emissions control policies (Rizzo 2005). Estimated reductions in nonaccidental deaths assuming reductions in ozone over the entire year were very similar to estimates based on the warm season only, e.g., 1,690 deaths avoided (90% CI: 745, 2,634) compared with 1,694 (90% CI: 747, 2,639) for the warm season using the Bell et al. (2004) risk estimate (data not shown).

**Figure 2:**
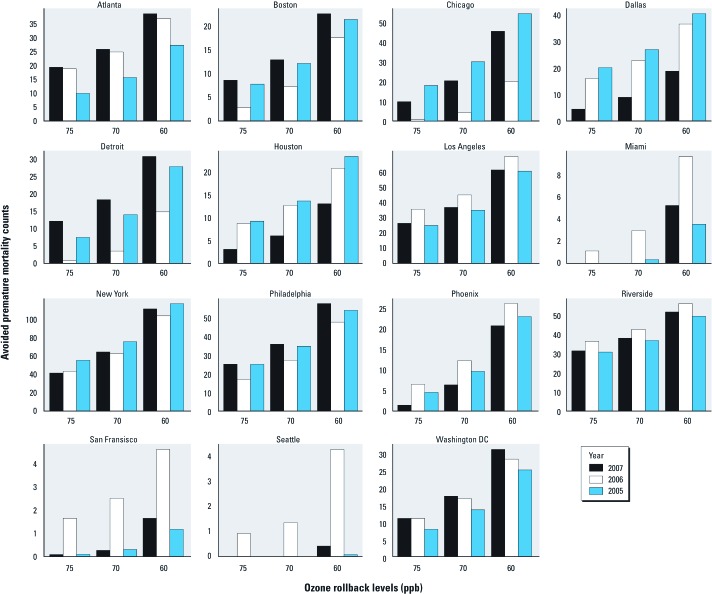
Estimated numbers of avoided nonaccidental deaths in 15 MSAs following regulatory attainments of 75, 70, and 60 ppb according to year (2005–2007) [estimated using national C-R functions from Bell et al. (2004)]. MSA population sizes are provided in Supplemental Material, Table S2 (http://dx.doi.org/10.1289/ehp.1104851).

*Avoided nonaccidental mortality by MSA.* Estimated numbers of avoided nonaccidental deaths for 15 MSAs suggested both geographic and temporal differences in ozone-related health effects (Figure 2). We calculated the most avoided deaths for New York and Los Angeles, which had the largest populations of the MSAs evaluated [for the MSA population sizes, see Supplemental Material Table S2 (http://dx.doi.org/10.1289/ehp.1104851)]. However, annual differences in MSA estimates are also related to spatial variations in the ozone concentrations observed across our air quality years (Figure 1). For example, Miami, which had the sixth largest population, had the third smallest estimate in reduced deaths, whereas estimated avoided deaths for Riverside (California), which had the 13th largest population, were comparable to estimates for Philadelphia, the fourth largest MSA. Temporal patterns of estimated effects also varied among MSAs. For example, we estimated twice as many deaths avoided in Seattle, Miami, and San Francisco during 2006 compared with 2005 or 2007, but a 50% decrease in premature mortality in Chicago and Detroit. Estimates for 2005 suggested major reductions in mortality would have occurred in the central U.S. cities of Chicago, Dallas, and Houston, whereas smaller impacts were estimated for Atlanta, Seattle, and Washington, DC.

## Discussion

The goal of our analysis was to estimate the health benefits that would be achieved by meeting the current ozone NAAQS, and additional benefits that might accrue from meeting more stringent standards. The HIA approach is based on research linking ozone exposures to adverse health effects, such as studies that indicate associations of a 10-ppb increase in daily ozone with a 0.87% increase in total mortality (Bell et al. 2004) and a 4.0% increase in respiratory mortality (Jerrett et al. 2009). Our results quantify the immediate health impacts of achieving ozone standards, instead of projecting benefits into the future. These results are of particular public health significance in light of the executive decision to withdraw proposed ozone NAAQS in the range of 70–60 ppb (Office of the Press Secretary 2011).

Hubbell et al. (2005) estimated that an 80-ppb ozone regulatory standard would result in 840 prevented mortalities for the United States based on 2000–2002 air quality data. Using more recent baseline incidence rates, population sizes, and estimated C-R functions, we estimated that a further reduction of 5 ppb from the current standard would result in nearly twice the number (approximately 1,900/year) of avoided all-cause mortalities. A 2008 U.S. EPA regulatory impact assessment for a proposed 65-ppb ozone standard suggested smaller overall health impact estimates than ours based on a 60-ppb standard (U.S. EPA 2008b). For example, the U.S. EPA estimated 450 avoided premature mortalities and 1.1 million avoided school-loss days based on a 65-ppb standard, whereas we estimated 1,700 avoided premature mortalities and 3.7 million avoided school-loss days under a 60-ppb standard. Geographic patterns of ozone concentrations estimated by Fann et al. (2011) were similar to patterns estimated for the present study, including areas of high ozone in southern California, the industrial Midwest, and portions of the South, including St. Louis and Atlanta.

In contrast with the present study, the U.S. EPA used CMAQ (Community Multiscale Air Quality) program modeling to project ozone levels to the year 2020, the expected date of full NAAQS compliance, assuming a series of increasingly stringent emissions reductions from future rules (U.S. EPA 2008b). In addition, the U.S. EPA assessment excluded the San Joaquin and South Coast Air Basins, significant population areas, because these regions are not expected to meet the 2020 projection (U.S. EPA 2008b). We used more recent data to estimate immediate health effects that would result from achieving standards, without assuming changes according to a regulatory compliance timeline. We also elected to use air monitor data to avoid the uncertainty associated with air quality modeling assumptions such as emissions inventories and chemical mixing (U.S. EPA 2008b). However, the use of monitor data has some disadvantages. For example, AQS distributions are heterogeneous and several regions are sparsely monitored, notably the Rocky Mountains [see Supplemental Material, Figure S1 (http://dx.doi.org/10.1289/ehp.1104851)]. Interpolated ozone concentrations in these areas have greater variability than locations proximate to other monitors, which may reduce the accuracy of estimated effects in these locations.

The use of 3 years of air quality data follows the U.S. EPA ozone NAAQS (U.S. EPA 2008c), while highlighting the temporal variability observed in the annual ozone exceedance. For example, we estimated substantial reductions in ozone levels for Houston in 2005 and 2006, but the reduction would have been much smaller in 2007. Atypical temperature and precipitation during the sampling season may partially explain this, highlighting the regional impact of weather on air pollutant concentrations. Also, we estimated reductions in premature mortality per MSA using a national risk function estimate. A preferred approach would be to apply appropriate city-specific estimates (Hubbell et al. 2009), but this would have been difficult to do given the data and analytical platform used for our analysis.

Avoided respiratory-related ED visits were estimated using C-R functions with varying population age ranges. A potential concern is that pooling estimates where some age groups are more heavily represented than others may generate biased estimates; however, pooling improves geographic coverage and provides more robust results at the national level. The MSA assessment might be improved by using population weighted results because it is difficult to separate effects due to the number of residents from effects due to ozone reduction. However, because our goal was to demonstrate overall health benefits, absolute numbers of prevented outcomes are a more relevant metric for assessing public health impacts compared to normalized estimates.

Based on our analysis, a 60-ppb ozone standard would result in the greatest numbers of avoided health effects. However, attainment of a 60-ppb standard, which is close to the non-anthropogenic background level of 40 ppb, would likely present significant challenges for regulatory interventions, and a less stringent alternative NAAQS standard of 70 ppb would also yield significant improvements to human health. On the other hand, implementation of tighter emissions regulation is important because ambient ozone levels are predicted to rise with changes in global climate (Chang et al. 2010) and preliminary studies suggest that the adverse health effects of ozone may be increased in warmer temperatures (Foster et al. 2000). Reductions in ambient ozone and related health benefits would also result in substantial economic benefits that were not considered in our analysis. Though the statutory requirements of the CAA preclude consideration of economic factors in setting the NAAQS, attainment costs are considered during the implementation phase of air pollution control policy decisions.

Although there is substantial consistency across epidemiological and clinical studies regarding the evidence of adverse health consequences associated with exposure to ambient ozone, uncertainty remains regarding the dose–response relationship, especially at low ozone concentrations. It is not clear from the published literature whether the biological processes driving short-term and long-term induced mortality are the same. Therefore, it is currently unknown whether premature deaths observed in long-term cohort studies are clinically unique from those observed in short-term time-series studies. This disparity may lead to a potential overestimation of ozone-linked mortality. Additionally, because the U.S. EPA’s current science assessment (U.S. EPA 2008c) identifies no threshold for the relationship between ozone exposure and premature mortality, our assessment underestimates the overall avoided premature mortality in regions where ozone concentrations fall below the assumed background level of 40 ppb.

By restricting our analysis to the ozone season, 1 May–30 September, we may have underestimated the magnitude of ozone-related health effects in regions with longer peak ozone seasons, including southern California and Texas. However, national estimates that were not restricted to the ozone season were comparable to those reported. Geographic underestimation may also have occurred in rural regions that lack sufficient monitors but are exposed to biogenic volatile organic compounds from terrestrial plant sources (Fiore et al. 2005).

There were also statistical and methodological limitations related to the software platform used in our analysis. Each step of risk assessment presents uncertainty, and it is difficult or impossible to comprehensively quantify the propagated effect of uncertainty introduced at multiple points in the analysis (Briggs et al. 2009; Levy et al. 2001). Our model did not account for uncertainty related to the ozone interpolation, the incidence rate estimates, and the annual population estimates from 2000 census data. Instead, our confidence intervals were based solely on variance from the health studies, which may have led us to underestimate overall uncertainty and led to potentially spurious conclusions. As suggested by Davidson et al. (2007), health effects estimates should be interpreted not necessarily as actual benefits, but as representations of the anticipated magnitude. One approach to addressing these limitations would be to utilize more sophisticated methods such as probabilistic-based spatial interpolation, localized risk estimates, or Bayesian models. This will be investigated in future research.

## Conclusions

Using national air monitor data and published C-R functions, we estimated health benefits from compliance with the current ozone NAAQS and potential benefits from adopting stricter ozone standards. A minimum ozone threshold associated with no increased risk of premature mortality has not been identified (Bell et al. 2006; Gryparis et al. 2004; Ito et al. 2005; Levy et al. 2005) and we contend that a more stringent standard would prevent substantial numbers of adverse health outcomes. Our findings suggest that attainment of the 75-ppb ozone NAAQS would prevent > 1,000 annual premature mortalities nationally, with substantial additional health benefits afforded from stricter air quality standards of 70 and 60 ppb. The potential impact of estimated health benefits varied by region and time over several years of data, but our analysis indicates that reducing ozone concentrations to levels proposed by the CASAC would result in dramatic public health benefits.

## Supplemental Material

(508 KB) PDF
