# Supplementary material for: Health Benefits from Large-Scale Ozone Reduction in the United States
Source: Environ Health Perspect. 2012 Jul 18;120(10):1404–10. doi: 10.1289/ehp.1104851 (PMC3491929; doi:10.1289/ehp.1104851)
Supplement: (508 KB) PDF [file ehp.1104851.s001.pdf]

SUPPLEMENTAL MATERIAL

TITLE: "Health Benefits from Large Scale Ozone Reduction in the United States"

AUTHORS: Jesse D Berman, Neal Fann, John W Hollingsworth, Kent E Pinkerton, William N Rom, Anthony M Szema, Patrick N Breysse, Ronald H White, and Frank C Curriero.

TABLE OF CONTENTS

Supplemental Material, Table S1……………………………………………………….. page 2

Supplemental Material, Table S2……………………………………………………….. page 3

Supplemental Material, Figure S1…...…………………………………………………….. page 4

Supplemental Material, References…..…………………………………………………….. page 5

Supplemental Material, Table S1. Exposed population sizes for the contiguous United States by health outcome

	U.S. Population (by Year)[1]			
**Age Group**	**2005**	**2006**	**2007**	**Related Health Outcomes**
Total Population	293,495,000	297,114,000	300,029,000	Mortality (non-accidental, all cause, cardiopulmonary); ER visits (respiratory)
0-2	8,139,000	8,240,000	8,328,000	Hospital Admissions (respiratory)
5-17	52,611,000	52,708,000	52,789,000	School Loss Days
5-34	121,586,000	122,237,000	122,895,000	Emergency Room Visits (respiratory)
18-65	184,325,000	187,165,000	189,167,000	Acute Respiratory Symptoms
30+	171,555,000	174,005,000	175,960,000	Mortality (respiratory)
65+	72,660,000	73,561,000	74,823,000	Hospital Admissions

[1] Population data was from the 2000 U.S. census and extrapolated to years 2005-2007 using growth factors (Woods & Poole Economics Inc. 2001). Values represent the population of exposed individuals for each health outcome

Supplemental Material, Table S2. Exposed population sizes for the 15 largest Metropolitan Statistical Areas (MSA's)

	U.S. Population (by Year)[1]		
**MSA**	**2005**	**2006**	**2007**
New York City, NY	18,683,000	18,823,000	18,919,000
Los Angeles, CA	12,867,000	12,967,000	13,034,000
Chicago, IL	9,409,000	9,502,000	9,570,000
Dallas, TX	5,768,000	5,894,000	5,991,000
Philadelphia, PA	5,809,000	5,849,000	5,874,000
Miami, FL	5,388,000	5,504,000	5,605,000
Houston, TX	5,242,000	5,371,000	5,444,000
Washington, DC	5,174,000	5,265,000	5,341,000
Atlanta, GA	4,874,000	4,977,000	5,058,000
Detroit, MI	4,478,000	4,499,000	4,510,000
Boston, MA	4,397,000	4,429,000	4,450,000
San Francisco, CA	4,131,000	4,176,000	4,211,000
Riverside, CA	3,876,000	3,962,000	4,039,000
Phoenix, AZ	3,832,000	3,927,000	4,013,000
Seattle, WA	3,189,000	3,234,000	3,272,000

[1] Values were extracted from the national population and used to examine the subsets of avoided premature mortality corresponding to each MSA

Supplemental Material, Figure S1. Distribution of ozone air monitors from 2005 to 2007 used in the BenMAP software (version 4.0) and county extents the 15 MSA's (U.S. Environmental Protection Agency 2005)

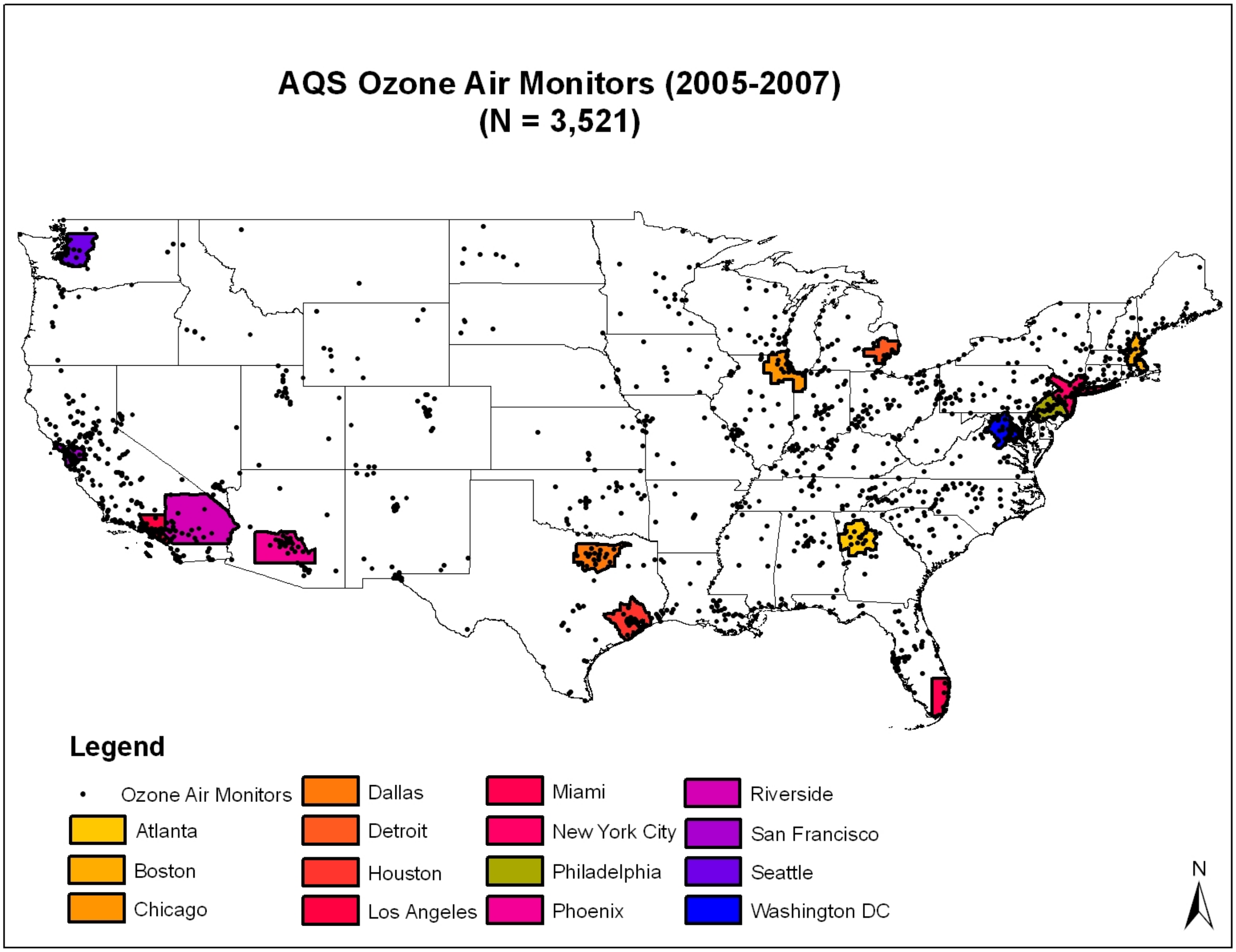

Supplemental Material, References

U.S. Environmental Protection Agency. 2005. Precision and Accuracy Criteria Pollutant Quality Indicator Summary Data Extraction (AMP255).

Woods & Poole Economics Inc. 2001. Population by Single Year of Age CD.
